# Unrestricted evening use of light‐emitting tablet computers delays self‐selected bedtime and disrupts circadian timing and alertness

**DOI:** 10.14814/phy2.13692

**Published:** 2018-05-22

**Authors:** Evan D. Chinoy, Jeanne F. Duffy, Charles A. Czeisler

**Affiliations:** ^1^ Sleep Health Institute and Division of Sleep and Circadian Disorders Departments of Medicine and Neurology Brigham and Women's Hospital Boston Massachusetts; ^2^ Division of Sleep Medicine Department of Medicine Harvard Medical School Boston Massachusetts; ^3^ Present address: Sleep and Fatigue Research Laboratory Warfighter Performance Department Naval Health Research Center San Diego California

**Keywords:** chronobiology, melatonin, sleep

## Abstract

Consumer electronic devices play an important role in modern society. Technological advancements continually improve their utility and portability, making possible the near‐constant use of electronic devices during waking hours. For most people, this includes the evening hours close to bedtime. Evening exposure to light‐emitting (LE) devices can adversely affect circadian timing, sleep, and alertness, even when participants maintain a fixed 8‐hour sleep episode in darkness and the duration of evening LE‐device exposure is limited. Here, we tested the effects of evening LE‐device use when participants were allowed to self‐select their bedtimes, with wake times fixed as on work/school days. Nine healthy adults (3 women, 25.7 ± 3.0 years) participated in a randomized and counterbalanced study comparing five consecutive evenings of unrestricted LE‐tablet computer use versus evenings reading from printed materials. On evenings when using LE‐tablets, participants' self‐selected bedtimes were on average half an hour later (22:03 ± 00:48 vs. 21:32 ± 00:27 h; *P* = 0.030), and they showed suppressed melatonin levels (54.17 ± 18.00 vs. 9.75 ± 22.75%; *P* < 0.001), delayed timing of melatonin secretion onset (20:23 ± 01:06 vs. 19:35 ± 00:59 h; *P* < 0.001), and later sleep onset (22:25 ± 00:54 vs. 21:54 ± 00:25 h; *P* = 0.041). When using LE‐tablets, participants rated themselves as less sleepy in the evenings (*P* = 0.030) and less alert in the first hour after awakening on the following mornings (*P* < 0.001). These findings demonstrate that evening use of LE‐tablets can induce delays in self‐selected bedtimes, suppress melatonin secretion, and impair next‐morning alertness, which may impact the health, performance, and safety of users.

## Introduction

Recent studies have demonstrated that use of light‐emitting (LE) electronic devices in the evening close to bedtime negatively affects physiology and behavior, especially that of sleep and circadian rhythms (Bonnefond et al. 2006; Van den Bulck 2007; Cajochen et al. 2011; Munezawa et al. 2011; Arora et al. 2013, 2014; Czeisler 2013; Foley et al. 2013; Wood et al. 2013; Gamble et al. 2014; Heath et al. 2014; Chang et al. 2015; Carter et al. 2016; Czeisler and Shanahan 2016). Rapid technological advances and market pressures have led to near ubiquitous use of LE‐devices (e.g., desktop, laptop, and tablet computers, cell/smart phones, televisions, and video games) in the modern home and workplace; and such devices are now integral to many daily functions including communication, commerce, recreation, and access to news and information. Greater utility, availability, and portability of LE‐devices have additionally led to widespread LE‐device use in the evening and incorporation into the bedtime routine. A recent National Sleep Foundation survey found that 90% of Americans reported use of LE‐devices within an hour of their bedtimes, and greater use was associated with worse sleep outcomes at all ages (Gradisar et al. 2013). That and other studies have shown that adolescents and young adults are particularly likely to use LE‐devices before bedtime, often multiple LE‐devices at the same time, and the amount of LE‐device use in the evening is associated with multiple negative outcomes including delayed bedtimes, longer sleep latencies, sleep interruption from the LE‐devices during the night, shorter sleep durations, increased daytime sleepiness, and even obesity (Van den Bulck 2007; Munezawa et al. 2011; Arora et al. 2013, 2014; Foley et al. 2013; Gradisar et al. 2013; Gamble et al. 2014; Pourzanjani et al. 2015; Carter et al. 2016; Czeisler and Shanahan 2016).

Light exposure *per se* is thought to cause many of the negative effects from evening use of LE‐devices. Retinal photoreceptors transmit light information to the master circadian clock in the hypothalamus, the suprachiasmatic nucleus (SCN), which regulates secretion of the pineal hormone melatonin together with rhythms in physiology and behavior including sleep, metabolism, immunity, alertness, and performance (Goel et al. 2013; Scheiermann et al. 2013; Qian and Scheer 2016). In the evening hours around bedtime, light exposure suppresses melatonin secretion and causes a phase delay shift in circadian rhythm timing such that melatonin secretion is reset to begin at a later time on subsequent nights (an effect similar to jet lag from extending daylight exposure later due to westward travel) (Khalsa et al. 2003; St Hilaire et al. 2012). This in turn can induce misalignment between the timing of the circadian rhythm of sleep propensity and the timing of sleep, reducing the duration, and quality of sleep (Czeisler et al. 1980; Dijk and Czeisler 1994). Misalignment can also cause delays in bedtime which, especially in children and adolescents, are associated with worse outcomes including behavioral risk factors and mental health problems such as depression (Gangwisch et al. 2010; Lemola et al. 2015; McGlinchey and Harvey 2015). There are also acute effects of light exposure that increase physiological and subjective levels of alertness (Lockley et al. 2006; Cajochen 2007; Rahman et al. 2014), which may impact when an individual feels tired and chooses to go to sleep when they are exposed to light in the evening. Furthermore, the effects of light on sleep, alertness, and circadian physiology are even greater from light exposures that are brighter, longer in duration, timed later in the evening, and contain short‐wavelength light (Lewy et al. 1980; Zeitzer et al. 2000; Brainard et al. 2001; Thapan et al. 2001; Khalsa et al. 2003; Lockley et al. 2006; Cajochen 2007; Santhi et al. 2012; St Hilaire et al. 2012; Lucas et al. 2014; Rahman et al. 2014). Because LE‐devices are often used for extended periods close to bedtime and their screens are typically illuminated by light‐emitting diodes (LED) that are rich in short‐wavelength light (Chang et al. 2015; Gringras et al. 2015) to which the human circadian system is particularly sensitive (Brainard et al. 2001; Thapan et al. 2001; Lockley et al. 2003; Rüger et al. 2013), their use is likely to increase alertness and impact sleep.

Our group reported findings from a laboratory‐controlled, within‐subject, counter‐balanced, and randomized study in which the effects of light exposure from five consecutive evenings of reading on a LE‐tablet computer were compared to five consecutive evenings reading from printed books prior to a strictly imposed 10 pm bedtime, followed by an 8‐h scheduled sleep episode in total darkness (Chang et al. 2015). Compared to the evenings with printed books, the LE‐tablet reading condition caused significant melatonin suppression, circadian phase delay shifts, longer sleep latencies, reduced rapid eye movement (REM) sleep, increased alertness prior to bedtime, and lower next‐morning alertness. Those findings provided direct evidence that the light exposure from a tablet computer negatively affected sleep, circadian rhythms, and alertness under controlled laboratory conditions. In that study, the duration of evening reading sessions was set to four hours, bedtimes were fixed at 22:00, and in the LE‐tablet condition the device was placed at a fixed distance from participants and activities were restricted to reading electronic books (eBooks). Other studies that tested the effects of LE‐devices have similarly controlled aspects of the duration, timing, illuminance, and screen content of light exposures (Cajochen et al. 2011; Wood et al. 2013; Heath et al. 2014; van der Lely et al. 2015; Gronli et al. 2016). Now that the effects of LE‐devices on sleep, circadian rhythms, and alertness have been established even when bedtimes are held at a fixed time under controlled laboratory conditions, it is critical to evaluate the impact of LE‐devices on *self‐selected* bedtimes, sleep, alertness, and circadian rhythms in a randomized trial under controlled laboratory conditions.

Increased evening alertness, melatonin suppression, and delayed circadian rhythm timing are likely key factors contributing to epidemiological findings showing associations between evening LE‐device use and later bedtimes (Foley et al. 2013; Gamble et al. 2014; Hysing et al. 2015). Additionally, such devices have numerous capabilities (e.g., internet, email, social media, games, videos, calendar, live streaming). Personal engagement with the diverse available activities may contribute to the selection of later bedtimes, thereby further prolonging evening light exposure from the device, and potentially leading to similar or even greater effects on sleep and circadian rhythms as those already observed under conditions with fixed bedtimes and light exposures. Therefore, in a follow‐up investigation to Chang et al. (Chang et al. 2015) we utilized a similar study design under controlled laboratory conditions, while allowing participants to self‐select their nightly bedtimes and LE‐tablet activities. We aimed to test whether under these unrestricted conditions, evening use of LE‐tablets compared to reading printed materials would affect self‐selection of bedtimes, and influence melatonin secretion, sleep, circadian timing, and alertness.

## Methods

### Ethical approval

The protocol was conducted in accordance with the Declaration of Helsinki and was approved by the Partners HealthCare System Institutional Review Board. Participants gave written informed consent prior to study and were paid for their participation.

### Participants

Nine healthy adults (six men, three women) aged 25.7 ± 3.0 years (mean ± SD) participated in the study. Screening included health and sleep‐wake history; psychological questionnaires; clinical tests of blood and urine; electrocardiography; and physical, psychological, and ophthalmological examinations. Exclusion criteria included age <18 or >30 years, body mass index <18.5 or >29.9 kg/m^2^ (22.8 ± 2.6 kg/m^2^; mean ± SD), average self‐reported sleep duration <7 or >9 h, average bedtime outside of 21:00–24:00 and average wake time outside of 05:00–09:00, current or past medical or psychiatric disorders, excessive use of caffeine or alcohol, overnight or rotating shift work within 3 years prior to study, travel across 2 or more time zones within 3 months prior to study, eye injury or disease, colorblindness, and current use of drugs, nicotine, or medications (other than oral contraceptives). Female participants had to report having regular menstrual cycles (26–35 days).

### Prestudy conditions

Participants maintained a fixed sleep schedule of 22:00–06:00 for at least 1 week immediately prior to study. Adherence to the sleep schedule was verified via wrist actigraphy (Actiwatch‐L, Mini‐Mitter Respironics, Bend, OR), call‐ins to a time‐stamped voicemail at bed and wake times, and daily written sleep diaries. Participants were instructed to abstain from caffeine, alcohol, nicotine, and medications (other than prescribed oral contraceptives) for the week prior to study, and urine toxicology tests were completed at laboratory admission to verify compliance.

### Study protocol and lighting conditions

**Figure 1:**
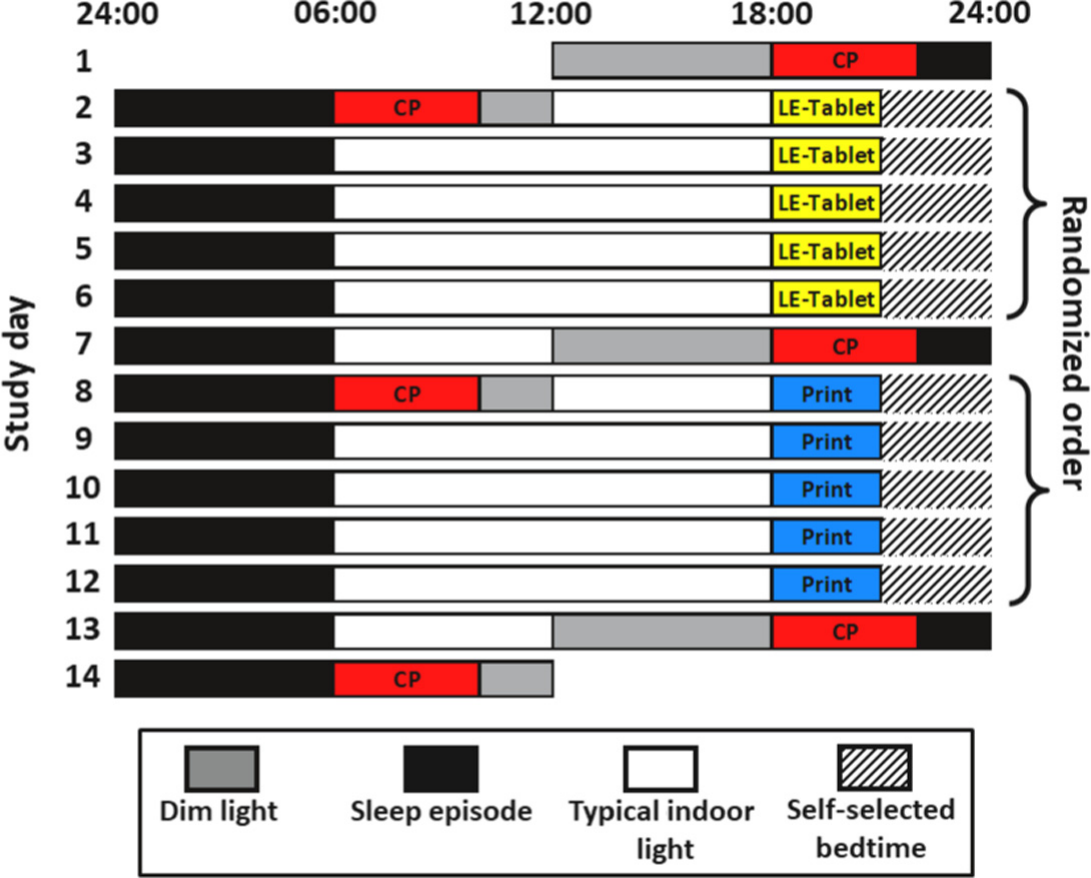
Study protocol. Representative raster plot of the 14‐day inpatient study protocol. Clock hour is indicated across the *x*‐axis and study day on the *y*‐axis. Two sets of five consecutive evening Print or LE‐tablet condition sessions in dim lighting occurred on Days 2‐6 and 8‐12. Conditions were counterbalanced and order was randomized. Evening sessions began at 18:00 and participants continuously engaged in the condition activities until a break time that started at 20:45. After the break, at 21:05 participants took a ~5‐min computerized test battery assessing sleepiness/alertness. Immediately following the test battery, participants were able to self‐select their bedtime (denoted by hatched bars). Black bars denote sleep episodes in darkness, which were scheduled from 22:00 to 06:00 on Nights 1, 7, and 13. Constant posture (CP) procedures in dim lighting, denoted by red bars, occurred on Days 1, 2, 7, 8, 13, and 14, during the 4 hours both immediately before (18:00–22:00) and after (06:00–10:00) the scheduled 22:00‐06:00 sleep episodes that began on Days 1, 7, and 13. Participants were exposed to dim lighting in times denoted by gray bars both before (12:00–18:00) and after (10:00–12:00) the CPs, and were exposed to typical indoor lighting during times denoted by white bars. See Methods for further study protocol details.

During the 14‐day inpatient laboratory protocol (Fig. 1), participants were studied individually and lived in light and sound‐attenuated research rooms at the Center for Clinical Investigation at Brigham and Women's Hospital. Lights in the rooms were ceiling‐mounted fluorescent lamps (T8 or T12 4,100 K lamps; Philips Lighting, Eindhoven, The Netherlands), and light measurements were taken using an IL1400 radiometer/powermeter with a SEL‐033/Y/W detector (International Light Inc., Peabody, MA). Participants were in dim room lighting (~0.0048 W/m^2^, ~1.8 lux measured at 137 cm from the floor facing toward the walls and had a maximum of 0.025W/m^2^, ~8.0 lux measured at 187 cm from the floor facing toward the ceiling anywhere in the room) during CPs and evening Print and LE‐tablet sessions, were in darkness during sleep episodes, and were in typical room lighting (~0.23 W/m^2^, ~89 lux measured at 137 cm from the floor facing toward the walls and had a maximum of ~0.48 W/m^2^, ~150 lux measured at 187 cm from the floor facing toward the ceiling anywhere in the room) at all other times.

The protocol was a randomized and crossover design that tested two conditions within each participant: (1) Print; five consecutive evenings of reading from printed materials, and (2) LE‐tablet; five consecutive evenings of using a LE‐tablet computer (iPad; first‐generation, Apple Inc., Cupertino, CA).

### Print and LE‐tablet session procedures

Two sets of five consecutive evening Print or LE‐tablet condition sessions occurred on Days 2–6 and 8–12 of the protocol (Fig. 1). Conditions were counter‐balanced and order was randomized. In the Print condition sessions, participants had to continuously read from printed materials (i.e., books, magazines, newspapers) and were free to switch their reading material at any time during the sessions. Participants brought their own reading materials to the laboratory and were additionally supplied with a new newspaper and a variety of magazines at the start of each Print session. In the LE‐tablet condition sessions, participants had to continuously use the LE‐tablet computer and their choice of activities on the device were unrestricted (i.e., participants could read eBooks, browse the internet, send emails, play computer games, watch videos, etc.) and they could change activities at any time. In both conditions there was a table placed in front of the participant and they were allowed to hold the reading material or the device at any comfortable distance from their eyes. Participants could change the distance they were holding the printed material or the device at any time, although in the LE‐tablet condition they had to use the device with the screen at an angle facing their eyes. A technician was present in the room with the participant during all sessions to ensure compliance, take light readings, and assist with the procedures.

Sessions began at 18:00 and participants were seated in a fixed location in the room and had to either read printed materials or use the LE‐tablet until a scheduled 15‐min break from 20:45 to 21:00. During the break, participants were instructed to not read or use the device but they could walk around the room, use the bathroom, talk to the technician, or do other sedentary activities. After the break, participants were seated in bed in a semirecumbent position and at 21:05 took a scheduled ~5‐min computerized test battery assessing sleepiness/alertness. Immediately following the test battery, the technician informed the participant that they could choose to either keep reading (or keep using the device, depending on the condition) or to go to sleep. If the participant chose to go to sleep then the technician prepared the room and the participant for sleeping conditions (the bed was lowered to a fully recumbent position and lights were turned off) within 10 min. Instead, if the participant chose to continue with the session, then they immediately continued reading or using the device and were also told by the technician that they could choose to end the session to go to sleep at any time by informing the technician. Throughout the sessions, participants had knowledge of clock time from a digital clock with a nonilluminated display that was placed within their view. During the LE‐tablet sessions the participants additionally could view the clock from the device's display.

During all Print and LE‐tablet sessions the participants were in continuous dim room lighting until their self‐selected bedtime when lights were turned off. There was no additional lighting in the room during Print sessions but on LE‐tablet sessions the participants were exposed to the additional lighting from the device. Light measurements were taken and recorded by a technician at the beginning and end of each session, at the top of every hour during each session, and every time the participant switched the printed material they were reading or changed their activity on the device. Light readings were taken with a sensor held next to the participant's eye and pointed along their angle of gaze at the printed material or the device. In our previous study (Chang et al. 2015), the LE‐device was placed on a stand at a fixed distance from the participant's eyes and illuminance readings were used to confirm they were exposed to light between 30 and 50 photopic lux. In this study, photopic lux illuminance measured from light readings in the LE‐tablet sessions averaged 38.4 ± 23.2 lux (mean ± SD, range: 1.5–148.2 lux) and in the Print sessions was 0.7 ± 0.2 lux (mean ± SD, range: 0.2–1.4 lux). The average approximate irradiance during the LE‐tablet sessions was 0.13W/m^2^. We used an example of a screen with the average photopic lux in order to calculate the retinal photopigment‐weighted illuminance measures, which were: 41.43 Cyanopic lux, 40.31 Melanopic lux, 40.37 Rhodopic lux, 39.63 Chloropic lux, and 37.74 Erythropic lux. The spectral power distribution of the LE‐tablet used in the study was reported in Figure 4 in our previous study (Chang et al. 2015).

### Constant posture (CP) procedures

CP procedures occurred on Days 1–2, 7–8, and 13–14, during the 4 h immediately before and after the scheduled 22:00–06:00 sleep episodes that began on Days 1, 7, and 13 (Fig. 1). These were the days before and after each 5‐day Print or LE‐tablet condition. During CPs participants were in dim room lighting, were limited to performing only sedentary activities while remaining awake in a semirecumbent posture in bed, and were continuously monitored by technicians sitting inside the room to ensure compliance.

### Melatonin and circadian phase assessments

Blood samples were taken via an indwelling forearm IV catheter every hour during CPs and on the fifth night of each Print or LE‐tablet session. Sample collection times were Day 1 16:00 – Day 2 10:00, Day 6 18:00 – Day 8 10:00, and Day 12 18:00 – Day 14 10:00. Plasma was frozen and assayed for melatonin at Solidphase, Inc. (Portland, ME) via direct plasma melatonin radioimmunoassay (Bühlmann Melatonin RIA, ALPCO, Salem NH).

Circadian phase was assessed using the dim‐light melatonin onset (DLMO), calculated as the clock time that melatonin levels rose above 25% of the peak‐to‐trough amplitude determined by a 3‐harmonic fit of the melatonin data from the baseline CP on Days 1‐2. Circadian phase shifts were calculated as the difference in clock time of DLMO during the CPs immediately preceding and succeeding each 5‐day condition (i.e., Day 7 DLMO minus Day 1 DLMO and Day 13 DLMO minus Day 7 DLMO). Melatonin area‐under‐the‐curve (AUC) was assessed using the trapezoidal method applied to the data from the fifth session in each condition, from the start of the session (18:00) until the break time (20:45). Melatonin suppression was calculated as the percent change in melatonin AUC from the fifth session in each condition compared to the corresponding time interval during the CP on the following evening when the participant remained in dim light.

### Sleep and wakefulness EEG assessments

PSG data were obtained with digital recorders (Vitaport‐3, Temec Instruments B.V., Kerkrade, The Netherlands) on the CP nights and the fifth nights in each condition. Electrodes were applied prior to the CPs and the Print and LE‐tablet evening sessions to record electroencephalography (EEG) from brain sites referenced to contra‐lateral mastoid processes for F3‐M2, F4‐M1, C3‐M2, C4‐M1, O1‐M2, and O2‐M1, left and right electrooculograms (EOG), and submental electromyograms (EMG). Signal impedances were <10 k ohms at the start of the recording, and PSG data were recorded at 256 Hz.

Sleep stages (N1, N2, N3, and REM) and wakefulness were manually scored from the PSG data in 30‐sec epochs using standard criteria (Iber et al. 2007). Sleep measures included time‐in‐bed (time between lights off and lights on), total sleep time (TST; total time spent in any sleep stage), sleep efficiency (TST divided by time‐in‐bed), sleep onset latency (SOL; time from lights off until the first occurrence of three consecutive sleep epochs), sleep onset (clock time of sleep onset), and wakefulness after sleep onset (WASO; time awake in each sleep episode following SOL).

To assess objective sleepiness during the fifth evening session of each condition, EEG spectral analysis using a fast Fourier transform (FFT) was applied to EEG data recorded during a 3‐min Karolinska Drowsiness Test (KDT) (Åkerstedt and Gillberg 1990) scheduled at 21:05. During the KDT, the participant was seated in a semirecumbent position in bed, was instructed to avoid moving or blinking, and to fix their gaze for 3 min on a dot on a piece of paper placed directly in front of them. EEG artifacts were visually identified and manually removed in 2‐sec epochs from KDT EEG data prior to spectral analysis. EEG data were low‐pass filtered at 30.0 Hz and high pass filtered with a time constant of 1.0 sec (equivalent to 0.159 Hz). Estimates of EEG power were output per 2‐sec epoch using a 10% cosine‐tapered window in 0.5‐Hz bins comprising the 0.5–20.0 Hz frequency range. EEG power in the 1.0–7.5 Hz bins was averaged for calculation of the delta/theta frequency range. KDT analyses were done separately for frontal, central, and occipital brain regions using data from brain sites F3‐M2, C3‐M2, and O1‐M2, respectively (in one participant there were significant EEG artifacts in those sites, and data from F4‐M1, C4‐M1, and O2‐M1 were used instead). For data analysis, the LE‐tablet condition EEG power was expressed as a percentage of the Print condition EEG power.

### Subjective sleepiness assessment

Subjective sleepiness was assessed with the Karolinska Sleepiness Scale [KSS, (Åkerstedt and Gillberg 1990)] once each evening (at 21:05, just prior to the KDT described above) and five times each morning at 10‐min intervals following the 06:00 wake time (scheduled at 06:01, 06:11, 06:21, 06:31, and 06:41). The KSS is a 9‐point Likert scale in which participants are asked to rate their level of alertness/sleepiness over the preceding 5 min. The KSS was administered using a computer while participants were seated in a semirecumbent position in bed.

### Daytime procedures

When not taking alertness or performance tests, during the daytime participants had free time. They were allowed to move about their study room, talk to the study staff members, and engage in sedentary activities such as reading, hobbies, or watching movies. Subjects were not allowed to nap or exercise, and did not have access to the LE‐tablet or other LE‐devices during the daytime (including their personal phone, which they did not have access to at any point during the study).

### Statistical analysis

Paired *t*‐tests were used to determine differences between conditions for self‐selected bedtimes, melatonin suppression, DLMO clock hour and phase shift, EEG power spectra and delta/theta power, and PSG sleep architecture measures. Shapiro–Wilk tests were used to confirm that data were normally distributed. KSS data were not normally distributed and were therefore analyzed with general linear mixed models fit with multinomial distribution and with Participant as a random effect. Factors Condition (Print vs. LE‐tablet) and Time (06:01, 06:11, 06:21, 06:31, 06:41) were analyzed for morning KSS data, and Condition was analyzed for evening KSS data. Condition order and interaction effects were tested but were nonsignificant and were therefore removed from the final model. Correlations between average duration of LE‐tablet use and DLMO phase shift were calculated with Pearson correlation coefficients. Effect sizes for paired *t*‐tests were calculated with Cohen's d (using the Cohen's d_z_ calculation), and effect sizes for factors in general linear mixed models were calculated with partial eta‐squared (*η* ^2^_p_) (Lakens 2013). Data are presented as mean ± SD. *P*‐values of alpha <0.05 were considered significant. Statistical analyses were performed with SAS (version 9.3, SAS Institute Inc., Cary, NC).

Data from one female participant were excluded from the melatonin suppression analysis because of missing blood samples during their fifth Print condition evening session. Additionally, data from two participants (one female and one male) were excluded from the evening KDT EEG power spectral analyses because of EEG artifacts present throughout PSG recordings during their fifth Tablet condition evening sessions.

## Results

### Self‐selected bedtimes

**Figure 2:**
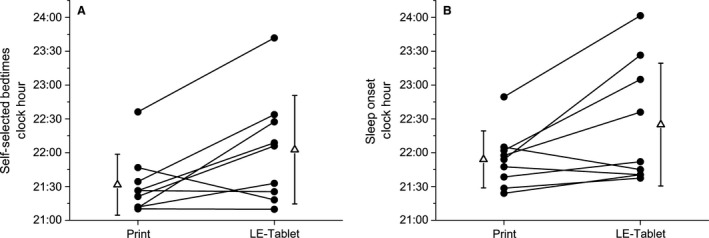
Self‐selected bedtimes and sleep onset times. Clock hour of (A) average self‐selected bedtime for all Print and LE‐tablet condition nights and (B) sleep onset during the fifth Print and LE‐tablet condition nights. In both panels, individual participant data are shown in black circles and condition group data (mean ± SD) are shown in open triangles beside the individual participant data. In (A), participants self‐selected significantly later average bedtimes on LE‐tablet nights compared to Print nights (Print: 21:32 ± 00:27 h; LE‐tablet: 22:03 ± 00:48 h, *t*_8_ = 2.63; *P* = 0.030; *d* = 0.88). In (B), participants fell asleep significantly later on the fifth LE‐tablet condition night compared to the fifth Print condition night (Print: 21:54 ± 00:25 h; LE‐tablet: 22:25 ± 00:54 h, *t*_8_ = 2.43; *P* = 0.041; *d* = 0.81).

Participants self‐selected significantly later average bedtimes on LE‐tablet nights compared to Print nights (LE‐tablet: 22:03 ± 00:48 h, Print: 21:32 ± 00:27 h; *t*_8_^ ^= 2.63; *P* = 0.030; *d* = 0.88; Fig. 2A).

### Melatonin suppression and circadian phase

**Figure 3:**
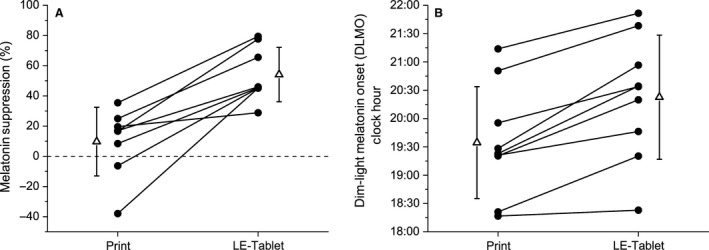
Melatonin suppression and circadian phase. (A) Melatonin suppression and (B) clock hour of dim‐light melatonin onset (DLMO) for Print and LE‐tablet conditions. In both panels, individual participant data are shown in black circles and condition group data (mean ± SD) are shown in open triangles beside the individual participant data. In (A), melatonin suppression is expressed as the percent change in melatonin area‐under‐the‐curve (AUC) from 18:00 to 20:45 during the fifth session in each condition compared to the corresponding time interval during the CP (shown as dashed horizontal line at zero) on the following evening. Melatonin levels were significantly suppressed on the fifth LE‐tablet session compared to the fifth Print session (Print: +9.75 ± 22.75%; LE‐tablet: +54.17 ± 18.00%, *t*_7_ = 5.73; *P* < 0.001; *d* = 2.03). In (B), DLMO circadian phase was significantly later during the CP following the LE‐tablet condition compared to the CP following the Print condition (Print: 19:35 ± 00:59 h; LE‐tablet: 20:23 ± 01:06 h, *t*_8_ = 5.84; *P* < 0.001; *d* = 1.95).

**Figure 4:**
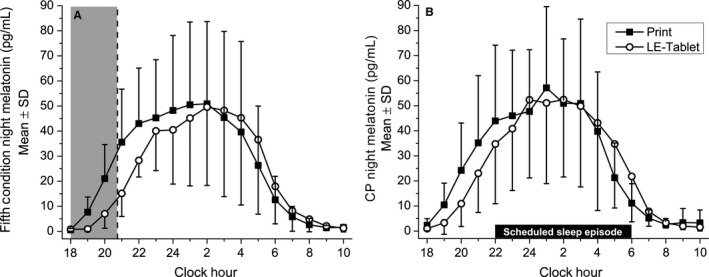
Plasma melatonin levels on condition and constant posture (CP) nights. Absolute melatonin levels from hourly blood plasma samples taken during (A) the fifth night in each condition and (B) the CP night following each condition. In both panels, condition group data (mean ± SD) for the Print condition are depicted in black squares and for the LE‐tablet condition are depicted in open circles. Clock hour is indicated across the *x*‐axis, and melatonin levels in pg/mL are depicted on the *y*‐axis. In (A), the shaded gray area from 18:00 to 20:45 indicates the time interval from the start of the evening session to the beginning of the break time, representing the minimum duration when all participants were still engaged in the session activities (and thus being directly exposed to light from the LE‐tablet) prior to being able to choose to go to sleep. In (B), sleep episodes were scheduled from 22:00 to 06:00 and CP procedures (see Methods for details) took place during the four hours before (18:00–22:00) and after (06:00–10:00) the CP scheduled sleep episode. Melatonin area‐under‐the‐curve (AUC) was assessed during the 18:00–20:45 intervals in (A) and (B), and AUC data within each condition and between nights were compared for the analysis of melatonin suppression. Circadian phase and phase shifts (see Methods for details) were assessed using the dim‐light melatonin onset (DLMO) on the CP nights in (B).

Melatonin levels were suppressed significantly more on the fifth LE‐tablet session compared to the fifth Print session (LE‐tablet: +54.17 ± 18.00%, Print: +9.75 ± 22.75%; *t*_7_ = 5.73; *P* < 0.001; *d* = 2.03; Figs. 3A and 4A). Circadian phase assessed by the dim‐light melatonin onset (DLMO) was significantly later following the LE‐tablet condition compared to the Print condition (LE‐tablet: 20:23 ± 01:06 h, Print: 19:35 ± 00:59 h; *t*_8_ = 5.84; *P* < 0.001; *d* = 1.95; Figs. 3B and 4B). Additionally, the magnitude of DLMO phase shift was significantly different between conditions, with phase advance shifts in the Print condition and phase delay shifts in the LE‐tablet condition (Print: +39.20 ± 45.86 min; LE‐tablet: ‐33.36 ± 25.27 min, *t*_8_ = 4.36; *P* = 0.002; *d* = 1.45). While the duration of light exposure from the LE‐tablet varied between participants and from evening‐to‐evening in the LE‐tablet condition, the magnitude of DLMO phase shift in each participant was not significantly correlated with the average duration (across the five evenings) of LE‐tablet light exposure (*r* = −0.23, *P* = 0.553).

### Sleep architecture

Polysomnography (PSG) was recorded on the fifth night in each condition (Table 1), when participants self‐selected their bedtimes. Although time‐in‐bed was not significantly different between conditions (Print: 498.83 ± 23.52 min; LE‐tablet: 473.89 ± 55.47 min, *t*_8_ = 1.61; *P* = 0.146; Table 1), the clock hour when participants fell asleep was significantly later in the LE‐tablet condition (LE‐tablet: 22:25 ± 00:54 h, Print: 21:54 ± 00:25 h; *t*_8_^ ^= 2.43; *P* = 0.041; *d* = 0.81; Fig. 2B and Table 1). When we examined the structure of sleep on those nights, the fifth LE‐tablet condition night had significantly more minutes of stage N3 (LE‐tablet: 104.00 ± 43.28 min, Print: 94.17 ± 44.89 min; *t*_8_ = 2.89; *d* = 0.96; *P* = 0.020), and significantly fewer minutes of wakefulness after sleep onset (WASO; LE‐tablet: 20.61 ± 18.80 min, Print: 49.83 ± 33.58 min; *t*_8_ = 2.73; *d* = 0.91; *P* = 0.026) compared to the fifth Print condition night. No other PSG sleep architecture measure was significantly different between conditions (Table 1).

**Table 1:** Sleep architecture on the fifth night in each condition

Measure	Print	LE‐Tablet	*t*_df=8_	*P*
N1 (min)	34.28 ± 9.68	32.72 ± 13.85	0.37	0.719
N2 (min)	187.33 ± 29.60	183.28 ± 45.53	0.48	0.643
N3 (min)	94.17 ± 44.89	104.00 ± 43.28	***2.89***	***0.020***
REM (min)	120.33 ± 14.65	114.50 ± 19.75	0.67	0.524
Wakefulness (min)	62.72 ± 41.75	39.39 ± 23.16	1.72	0.123
Time‐In‐Bed (min)	498.83 ± 23.52	473.89 ± 55.47	1.61	0.146
TST (min)	436.11 ± 43.03	434.50 ± 49.09	0.11	0.914
Sleep efficiency (%)	87.48 ± 8.09	91.86 ± 4.55	1.70	0.127
SOL (min)	13.39 ± 11.70	19.28 ± 8.40	1.23	0.254
Sleep onset (clock hour)	21:54 ± 00:25	22:25 ± 00:54	***2.43***	***0.041***
WASO (min)	49.83 ± 33.58	20.61 ± 18.80	***2.73***	***0.026***

Sleep stages (N1, N2, N3, and REM) and wakefulness were manually scored from PSG data in 30‐sec epochs, recorded on the fifth night of the Print and LE‐tablet conditions when bedtimes were self‐selected by participants (see Methods for details of PSG data collection and analysis). Sleep architecture measures include sleep stages (minutes), time‐in‐bed (time between lights off and lights on), total sleep time (TST; total time in any sleep stage), sleep efficiency (TST divided by time‐in‐bed), sleep onset latency (SOL; time from lights off until the first occurrence of three consecutive sleep epochs), sleep onset (clock hour of sleep onset), and wakefulness after sleep onset (WASO; time awake in each sleep episode following SOL). Data are expressed as mean ± SD. Differences between conditions were analyzed with paired *t*‐tests, and statistical significance at *P* < 0.05 shown in bold and italics.

### Evening and morning sleepiness and alertness

**Figure 5:**
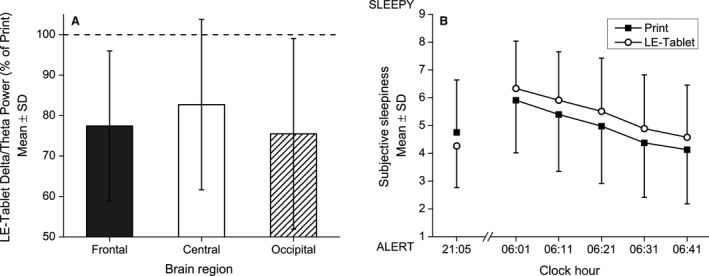
Delta/theta EEG power and subjective sleepiness ratings. (A) Evening delta/theta EEG power and (B) evening and morning subjective sleepiness ratings for Print and LE‐tablet conditions. In (A), delta/theta EEG power (inclusive of the 1.0–7.5 Hz range) for frontal, central, and occipital brain regions are expressed for the LE‐tablet condition as a percentage (mean ± SD) of the Print condition (shown as dashed horizontal line at 100%). EEG data were analyzed from the Karolinska Drowsiness Test (KDT) taken during the fifth evening session in each condition (see Methods for details of KDT protocol and EEG analysis). Evening delta/theta EEG power was significantly lower, indicating lower sleepiness/greater alertness, in the LE‐tablet condition for the frontal (77.42 ± 18.55%, *t*_6_ = 3.22; *P* = 0.018; *d* = 1.22) and occipital (75.49 ± 23.56%, *t*_6_ = 2.75; *P* = 0.033; *d* = 1.04) brain regions but only showed a non‐significant trend for being lower in the central brain region (82.70 ± 21.05%, *t*_6_ = 2.17; *P* = 0.073; *d* = 0.82). In (B), subjective sleepiness ratings are from the Karolinska Sleepiness Scale (KSS) taken once each evening (21:05) and five times each morning at 10‐min intervals following the 06:00 wake time (06:01, 06:11, 06:21, 06:31, and 06:41). Higher numbers on the KSS indicate ratings of greater sleepiness. Condition group data (mean ± SD) for the Print condition are depicted in black squares and for the LE‐tablet condition are depicted in open circles. Evening subjective sleepiness ratings were significantly lower in the LE‐tablet condition compared to the Print condition (Print: 4.76 ± 1.88; LE‐tablet: 4.27 ± 1.50, *F*_1,74_ = 4.90; *P* = 0.030; *η* ^2^_p_ = 0.06). Morning subjective sleepiness ratings showed significant effects of Condition (*F*_1,432_ = 18.45; *P* < 0.001; *η* ^2^_p_ = 0.04), with greater sleepiness on mornings following LE‐tablet evening sessions compared to mornings following Print evening sessions, and Time (*F*_1,432_ = 124.62; *P* < 0.001; *η* ^2^_p_ = 0.22), with sleepiness progressively decreasing every 10 min after lights were turned on each morning.

**Figure 6:**
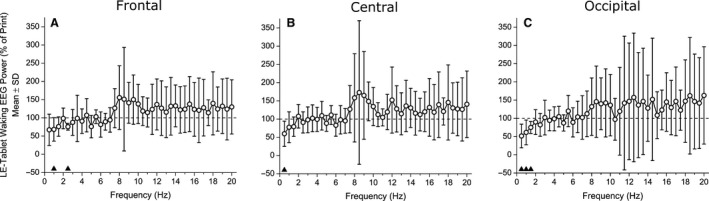
Evening waking EEG power spectral densities. Evening waking EEG power spectral densities in 0.5‐Hz bins comprising the 0.5–20.0 Hz frequency range for the (A) frontal, (B) central, and (C) occipital brain regions. Data are expressed for the LE‐tablet condition (in open circles) as a percentage (mean ± SD) of the Print condition (shown as dashed horizontal line at 100%). EEG data were analyzed from the Karolinska Drowsiness Test (KDT) taken during the fifth evening session in each condition (see Methods for details of KDT protocol and EEG analysis). Black triangles above the abscissa indicate bins that were significantly different (*P* < 0.05) between conditions.

Delta/theta EEG power (averaged over the 1.0–7.5 Hz range, an objective measure of sleepiness) during the fifth evening session was significantly lower in the LE‐tablet condition compared with the Print condition for the frontal (77.42 ± 18.55%, *t*_6_ = 3.22; *P* = 0.018; *d* = 1.22) and occipital (75.49 ± 23.56%, *t*_6_ = 2.75; *P* = 0.033; *d* = 1.04) brain regions, but this did not reach significance in the central brain region although the effect size was still large (82.70 ± 21.05%, *t*_6_ = 2.17; *P* = 0.073; *d* = 0.82; Fig. 5A). EEG power spectral density across the 0.5–20.0 Hz range on the fifth LE‐tablet evening, calculated as percent of the fifth Print evening, was significantly lower in the frontal brain region for the 1.0 Hz (*t*_6_ = 2.59, *P* = 0.041) and 2.5 Hz (*t*_6_ = 6.78, *P* < 0.001) bins, in the central brain region for the 0.5 Hz bin (*t*_6_ = 3.07, *P* = 0.022), and in the occipital brain region for the 0.5 Hz (*t*_6_ = 3.95, *P* = 0.008), 1.0 Hz (*t*_6_ = 3.01, *P* = 0.024), and 1.5 Hz (*t*_6_ = 3.97, *P* = 0.007) bins (Fig. 6). No other frequency bins were significantly different between the fifth Print and LE‐tablet evenings.

Evening subjective sleepiness ratings were significantly lower in the LE‐tablet condition compared to the Print condition (LE‐tablet: 4.27 ± 1.50, Print: 4.76 ± 1.88; *F*_1,74_ = 4.90; *P* = 0.030; *η* ^2^_p_ = 0.06; Fig. 5B). Subjective sleepiness ratings the next morning also showed significant effects of condition (*F*_1,432_ = 18.45; *P* < 0.001; *η* ^2^_p_ = 0.04; Fig. 5B), with greater sleepiness on mornings following LE‐tablet sessions compared to mornings following Print sessions. Morning subjective sleepiness also showed an effect of time since waking, (*F*_1,432_ = 124.62; *P* < 0.001; *η* ^2^_p_ = 0.22; Fig. 5B), with subjective sleepiness progressively decreasing every 10 min for the first 40 min after lights were turned on.

### Print and LE‐tablet session activities

During Print evening sessions participants predominantly chose to read from books (58.7 ± 41.4% of time in Print sessions). This was followed by time spent reading from newspapers (35.3 ± 35.7%) and magazines (5.9 ±11.8%). We grouped LE‐tablet evening session activities into seven categories of computer applications or internet websites. For almost one‐third of the time, participants chose to read eBooks (30.1 ± 27.6%). This was followed by watching videos (20.0 ± 20.7%), using social media websites (14.9 ± 15.2%), writing or reading emails (14.8 ± 11.6%), and playing games (10.0 ± 22.3%), with minimal time on news or informational websites (2.1 ± 2.7%). Participants also spent 8.1 ± 11.9% of the time engaged in other activities/websites that did not fit into the larger activity categories.

## Discussion

We found that unrestricted evening use of a LE‐tablet computer led to self‐selection of later bedtimes, and this was associated with suppression of the sleep‐promoting hormone melatonin, decreased evening sleepiness, delayed circadian rhythm timing, later sleep onset, and increased morning sleepiness compared to evenings with unrestricted use of only printed materials. We also found that on the fifth (final) LE‐tablet night there were changes in sleep structure (increased SWS and decreased WASO) that are likely to be related to increased homeostatic sleep pressure from the self‐selected shorter sleep episodes on the preceding nights. Whether it was the alerting effect of the light exposure from the LE‐tablet alone, or the more diverse and engaging LE‐device activities that led to the self‐selection of later bedtimes cannot be determined from this study, but the overall result was a delay in the decision to go to sleep resulting in prolonged evening wakefulness (and in turn prolonged exposure to the light from the LE‐tablet). Our findings of later self‐selected bedtimes and disrupted circadian signaling, sleep, and alertness patterns are consistent with findings from previous laboratory studies and indicate that LE‐device use in the evening has multiple impacts on physiology and behavior, potentially explaining findings from epidemiologic studies.

The young adults in our study chose to go to bed significantly later (on average 31 min later) on evenings when using LE‐tablet computers compared to evenings when they read from printed materials. This finding is consistent with epidemiological findings that show greater LE‐device use in the evening is associated with later bedtimes, especially in children and adolescents (Foley et al. 2013; Gamble et al. 2014; Hysing et al. 2015). Late bedtime is associated with higher risk of a number of negative health and behavioral outcomes (Gangwisch et al. 2010; Lemola et al. 2015; McGlinchey and Harvey 2015), and therefore the impact of LE‐device use on bedtime has important clinical implications. In fact, a half‐hour later bedtime and/or shorter sleep duration in adolescents is associated with increased daytime sleepiness, caffeine use, depression, and suicidal ideation (Gangwisch et al. 2010; Boergers et al. 2014). While we observed a half‐hour delay of bedtime with a large effect size, it is possible that the controlled laboratory conditions in our study may have attenuated the delay in bedtime on evenings when using the LE‐tablet. Although we instructed participants that there were no restrictions on their LE‐device activities, they were not in their normal home environments, did not have access to their mobile phones, could use only the (single) device we provided them, and knew their activities were being observed by the research staff. While participants engaged in a wide range of device activities, only ~30% of their LE‐tablet session time was spent engaged in activities involving personal online content (email and social media), less than what has been reported in other studies where many participants report multitasking using more than one LE‐device in the evening (Van den Bulck 2007; Cain and Gradisar 2010; Foley et al. 2013; Gradisar et al. 2013; Arora et al. 2014; Gamble et al. 2014; Hale and Guan 2015; Hysing et al. 2015; Lemola et al. 2015; Levenson et al. 2016).

Delayed bedtimes should consequently delay sleep onset times, and indeed we found that the clock time when participants fell asleep was similarly later on the final night in the LE‐tablet condition, matching the average bedtime delay. While PSG data collection in our study was limited to the fifth night in each condition, we would have expected to find similarly delayed sleep onset times on the other LE‐tablet condition nights, given the large effect size and that those bedtimes were also delayed. On the fifth LE‐tablet night, participants also showed significantly greater time spent in sleep stage N3 and less time awake than on the fifth Print night. These PSG findings indicate deeper and more consolidated sleep on the final LE‐tablet night, likely due to greater homeostatic sleep pressure accumulated from the sleep deficit on the four previous nights of self‐selection of later bedtimes (but fixed wake times). Previous PSG sleep architecture findings after evening LE‐device use have been mixed. In our previous study with fixed bedtimes and wake times, it look 10 min longer on average for participants to fall asleep and there was less overall REM sleep in the LE‐tablet condition (Chang et al. 2015), but we did not find significant impacts on other sleep stages or the amount of wakefulness during the scheduled sleep opportunity. In studies that tested one evening with either 30 min (Gronli et al. 2016), one hour (Heath et al. 2014), or two hours (Rangtell et al. 2016) of LE‐tablet use before bedtime, no sleep stage measures were altered, nor were they impacted in a study with three hours of gazing at a LED‐backlit desktop computer screen before bedtime (van der Lely et al. 2015). However, in the study with 30 min of LE‐tablet use before bedtime (Gronli et al. 2016), lower levels of slow wave activity and delta/theta EEG power were found within the first three hours of the sleep episode, indicating some altered PSG activity from the LE‐tablet. Thus, there is evidence from multiple studies that the sleep EEG can be altered following evening use of LE‐devices, although the exact EEG changes are not consistent between studies.

We found significant disruption of circadian signaling from evening use of an LE‐tablet; the typical evening rise of melatonin secretion was suppressed by more than half and the timing of the circadian rhythm onset was 48 min later and significantly delayed compared to the print condition. In our previous study in which participants used an LE‐tablet for four hours each evening over five consecutive evenings (Chang et al. 2015), we reported a similar amount of melatonin suppression but a circadian phase delay of about double the size. Although we found very large effect sizes for the circadian signaling outcomes in this study, it remains unclear why the magnitude of the circadian rhythm phase delay was smaller, but it may be due to the night‐to‐night variability in the duration of LE‐tablet use (which on some nights was less than four hours). Nevertheless, together our two studies clearly indicate that repeated use of LE‐tablets prior to bedtime both suppresses melatonin and delays circadian timing.

LE‐tablets and many other consumer LE‐devices are backlit by LEDs that emit short‐wavelength rich light (Cajochen et al. 2011; Chang et al. 2015; Gringras et al. 2015). Melanopsin‐containing retinal ganglion cells are especially sensitive to these short‐wavelength light stimuli, and when activated in the evening cause the suppression of pineal melatonin secretion and phase delays in circadian timing (Zeitzer et al. 2000; Brainard et al. 2001; Thapan et al. 2001; Lockley et al. 2006; Santhi et al. 2012; Lucas et al. 2014; Najjar et al. 2014). One potential countermeasure for attenuating the circadian disruption from evening LE‐device light exposure would be to filter out short‐wavelength light, which can be accomplished by utilizing software that changes the screen's emitted color temperature or using a filter or glasses that attenuate short‐wavelength light (Rahman et al. 2011; Wood et al. 2013; Heath et al. 2014; Gringras et al. 2015; van der Lely et al. 2015).

Evening alertness was enhanced by the LE‐tablet use in our study, as evidenced by both reduced subjective evening sleepiness and reduced delta/theta EEG power. This was associated with reduced alertness the following morning, consistent with the results from our and other previous studies (Cajochen et al. 2011; Chang et al. 2015; van der Lely et al. 2015; Gronli et al. 2016). Interestingly, another study found that prolonged exposure (6.5 h) to bright light during the daytime and a reduction in the duration of screen time during the evening might be effective in mitigating these adverse effects (Heath et al. 2014; Rangtell et al. 2016). The acute effects of light on evening alertness and EEG are well‐characterized (Lockley et al. 2006; Cajochen 2007; Rahman et al. 2014). The alterations of subjective and objective alertness in the evening in this study suggest that it is this alerting effect of light from the evening LE‐device use that results in delayed bedtime selection. This prolongs evening light exposure and reduces sleep duration, causing even further disruption to circadian signaling. Our finding of reduced next‐morning alertness likely results from delays in both sleep onset and circadian phase, together pushing the biological nighttime later and making it more likely that a fixed wake time for school or work would occur during the biological nighttime. This is also what occurs in social jetlag, when it is difficult to wake on Monday for school or work following a weekend when sleep (and circadian timing) was delayed (Roenneberg et al. 2012).

There were a number of limitations in this study. Although we aimed to make the conditions more similar to real‐world LE‐tablet use than in previous studies by not restricting what the LE‐tablet was used for, we did limit access to devices earlier in the day. Likewise, we did not allow participants to use a mobile phone, television, laptop, or other electronic device in the evenings, as is typical among adolescents and young adults who frequently multitask using different LE‐devices (Van den Bulck 2007; Foley et al. 2013; Gradisar et al. 2013; Arora et al. 2014; Gamble et al. 2014; Hale and Guan 2015; Hysing et al. 2015; Lemola et al. 2015; Levenson et al. 2016). Due to the small sample size we were not able to analyze the effects that each *type* of LE‐tablet activity had on the outcomes. Future studies should be designed to address this, as different types of activities and especially interactive activities may have differential effects on evening arousal, thereby leading to differential impacts on bedtimes and sleep disruption (Cain and Gradisar 2010). We also controlled the room lighting throughout the wake episodes and did not allow participants to go outside. This choice was made to test the direct effects of the LE‐device (and the light emitted) and thus we chose to minimize the impact of background lighting to levels that would not be expected to alter circadian signaling. As one recent study reported (Rangtell et al. 2016), extended daytime exposure to bright indoor light prior to two hours of evening LE‐tablet use may reduce the impact of LE‐tablet light exposure on evening melatonin levels. Thus, other daytime and evening light exposure patterns prior to and during LE‐device use should be tested to better understand the range of impacts from these devices. In this study, we set wake time at 06:00 every morning to mimic a typical weekday work or school schedule and required participants to awaken then. If we had instead allowed self‐selection of wake time (in addition to bedtime) and/or had not enforced a wake time, it is possible that participants would have awakened later. A regular morning wake time and the associated light exposure helps entrain the circadian timing system (Duffy and Wright 2005; Wright et al. 2013), therefore without a regularly imposed wake time we would have expected to see greater circadian timing delays than those we observed in the LE‐tablet condition. We recruited only healthy participants who had regular sleep patterns that were fairly early (for their age group) prior to study. Yet, adolescents and young adults show greater variability in health status and sleep habits, and many are evening chronotypes (Roenneberg et al. 2004), all factors that may influence selection of bedtime. In testing the effects of five consecutive nights of LE‐tablet or Print use we also limited the PSG and blood sampling to the final (5th) night, and so we were unable to measure any day‐to‐day changes or acute effects in the circadian and sleep outcomes. It is also possible that by beginning the LE‐tablet or Print session at 6 pm we influenced how “late” the participants chose to stay up, by becoming bored or fatigued using the device or reading material. Had we instead started the evening sessions closer to their habitual bedtimes they might have stayed up even later than observed, because the novelty of the online content they were checking (especially email and social media) would not have depleted by that time. We also did not allow participants to adjust the brightness setting on the LE‐tablet, and so devices that have automatic brightness settings or internal filters may have produced different results.

Findings from several recent studies demonstrate that sleep and circadian timing are indeed altered by the light exposure in our modern electrical constructed environments compared with natural environments without electrical lighting (Wright et al. 2013; de la Iglesia et al. 2015; Yetish et al. 2015; Stothard et al. 2017). Even though widespread electric lighting has been available in many areas for more than 100 years, LE‐device use is a relatively new phenomenon, and poses serious clinical concerns (Czeisler 2013). In particular, children and adolescents are especially vulnerable to sleep and circadian disturbances affecting development, and they show some of the highest use and most negative effects from LE‐device use in the evening and at night (Van den Bulck 2007; Foley et al. 2013; Gradisar et al. 2013; Arora et al. 2014; Gamble et al. 2014; Hale and Guan 2015; Hysing et al. 2015; Lemola et al. 2015; Levenson et al. 2016). In fact, a recent study found that many very young children (4 years and younger) have their own portable LE‐devices, and more than a quarter of parents use LE‐devices when putting their young children to bed at night (Kabali et al. 2015). Thus, while LE‐devices are important tools that have great utility and can satisfy both personal and societal needs, their widespread use and their potential for producing adverse behavioral and physiological effects means that additional studies are still warranted. In particular, studies examining physiological and behavioral effects of LE‐device use in children and adolescents to understand the potential developmental, academic, and psychological impacts of use, and studies of countermeasures (such as filters, bright light exposure earlier in the day, etc.) are needed to inform clinical recommendations.

## Conflict of Interest

E.D.C. and J.F.D. report no conflicts of interest. C.A.C. reports no conflicts of interest related to the present work, but declares he is/was a paid consultant to Bose, Boston Celtics, Boston Red Sox, Columbia River Bar Pilots, Institute of Digital Media and Child Development, Jazz Pharma, Merck, Purdue Pharma, Quest Diagnostics, Samsung, Teva, Vanda Pharmaceuticals, Inc., and V‐Watch/PPRS; has received lecture fees from Global Council on Brain Health/AARP, Integritas Communications Group, Maryland Sleep Society, National Sleep Foundation, and Zurich Insurance Company, Ltd.; holds equity in Vanda Pharmaceuticals, Inc.; receives research/education support from Cephalon, Mary Ann & Stanley Snider via Combined Jewish Philanthropies, Jazz Pharma, Optum, ResMed, San Francisco Bar Pilots, Schneider, Simmons, Sysco, Koninklijke Philips Electronics, Vanda Pharmaceuticals, Inc.; is/was an expert witness in legal cases, including those involving Bombardier, Columbia River Bar Pilots, Continental Airlines, Fedex, Greyhound, Purdue Pharma, UPS; serves as the incumbent of a professorship endowed by Cephalon; and receives royalties from McGraw Hill, Houghton Miflin Harcourt, and Philips Respironics for the Actiwatch‐2 & Actiwatch Spectrum devices. C.A.C.'s interests were reviewed and are managed by Brigham and Women's Hospital and Partners HealthCare in accordance with their conflict of interest policies.
